# Gecko CD59 Is Implicated in Proximodistal Identity during Tail Regeneration

**DOI:** 10.1371/journal.pone.0017878

**Published:** 2011-03-28

**Authors:** Yongjun Wang, Ruili Wang, Shengjuan Jiang, Weijuan Zhou, Yan Liu, Yingjie Wang, Qing Gu, Yun Gu, Yingying Dong, Mei Liu, Xingxing Gu, Fei Ding, Xiaosong Gu

**Affiliations:** 1 Key Laboratory of Neuroregeneration, Nantong University, Nantong, China; 2 College of Life Science, Anhui Science and Technology University, Fengyang, China; New York State Institute for Basic Research, United States of America

## Abstract

Several adult reptiles, such as *Gekko japonicus*, have the ability to precisely re-create a missing tail after amputation. To ascertain the associated acquisition of positional information from blastemal cells and the underlying molecular mechanism of tail regeneration, a candidate molecule CD59 was isolated from gecko. *CD59* transcripts displayed a graded expression in the adult gecko spinal cord with the highest level in the anterior segment, with a stable expression along the normal tail. After tail amputation, *CD59* transcripts in the spinal cord proximal to the injury sites increased markedly at 1 day and 2 weeks; whereas in the regenerating blastema, strong *CD59* positive signals were detected in the blastemal cells anterior to the blastema, with a gradual decrease along the proximodistal (PD) axis. When treated with RA following amputation, *CD59* transcripts in the blastema were up-regulated. PD confrontation assays revealed that the proximal blastema engulfed the distal one after *in vitro* culture, and rabbit-anti human CD59 antibody was able to block this PD engulfment. Overexpression of the CD59 during tail regeneration causes distal blastemal cells to translocate to a more proximal location. Our results suggest that position identity is not restricted to amphibian limb regeneration, but has already been established in tail blastema of reptiles. The CD59, a cell surface molecule, acted as a determinant of proximal–distal cell identity.

## Introduction

The regeneration of a missing structure in adulthood is found in several classes of vertebrates, including fish, amphibians and reptiles. The urodele amphibians, characterized by extensive regenerative ability, are capable of regenerating limbs, tail, jaws, lens, and small sections of the heart [1]–[7]. Comparatively, anuran tadpoles and some reptiles have an attenuated regenerative ability with preservation of tail reconstruction after amputation or autotomy [8], [9]. Re-creation of either limb or tail occurs from a proliferative zone, the blastema, in which mesenchymal stem cells dedifferentiate from internal tissues or migrate from satellite cells. The blastema retains positional identity, which is used to regenerate only correct elements. For example, a wrist blastema regenerates a hand, whereas a shoulder blastema results in an entire arm [10]. Transplantation experiments of limb blastema confirmed that proximodistal (PD) identities are already established in the earliest stages of blastema [11], and that blastemal cells are responsible for the measure of the positional information. Several assays, including blastema rotation, proximodistal blastema engulfment and grafting of distal blastema on proximal blastema, have suggested that PD identity of blastemal cells is encoded as a graded property, and expressed at the cell surface [12]–[14].

Retinoic acid (RA) proximalizes the positional identity of blastemal cells in the proximodistal (PD) axis of regenerating urodele limbs over the small range of RA concentrations about 2.5-fold [15], [16]. By screening the cDNA libraries constructed from the distal blastemas of newts treated with RA, da Silva et al. [17] identified the molecule involved in the PD positional memory, Prod1. The amino acid sequence of newt Prod1 contains the conserved motif CCXXXXCN-characteristic of the CD59/Ly-6 family of the three-finger protein (TFP) superfamily, and eight cystine residues aligned with ten cystine residues conserved in other mammalian CD59. The protein was originally regarded as an ortholog of CD59, which interfered with the assembling MAC by preventing the binding of C9 to the C5b-8 complex [18]. The newt Prod1 is located in the cell surface with a glycosylphosphatidylinositol (GPI) anchor, and implicated in the local cell-cell interactions mediating positional identity [17]. Overexpression of Prod1 caused distal blastemal cells to proximally shift and resulted in shortening or deletion of the lower arm structures, suggesting that Prod1 is a cell surface determinant of P–D cell identity [11]. Prod1 is expressed in a stable gradient along the axis in the cells of the adult limb [10]. It was hypothesized that these cells were precursors of blastemal cells and that they inherited the gradient expression of Prod1 after amputation [10]. Recent comparative analysis of the recombinant Prod1 3D solution structure to other known TFPs using phylogenetic techniques found that Prod1 was not a good match for any of the TFP families, including CD59 present in mammals [19], assuming that the role of Prod1 in encoding PD identity was restricted to the newt. However, the conclusion derived from sequence-structure bioinformatic analysis is necessarily limited by the absence of the complete sequence of an urodele genome. As an alternative, further functional verification on the positional identity of CD59 from phylogenetically adjacent species is advantageous in clarifying the association between the protein and Prod1.

Although the mechanisms of limb and/or tail regeneration were distinct in their blastemal cell lineage [8], [20]–[22], it is conceivable that cells in tail blastema also retain the positional information required to form a new complex tail consisting of skin, muscle, fat, cartilage and neural tissues. The blastemal cells should obtain instructions about where to reconstruct the missing structure, and to which tissues they should differentiate, in a manner similar to that occurs in embryo development and limb regeneration. Here, we cloned the CD59 cDNA from *Gekko japonicus* and investigated its implication in positional identity during tail regeneration. The functional evidence of CD59 in tail-regenerative reptiles is contributable to the homologous analysis of Prod1.

## Results

### Isolation and analysis of gecko *CD59*

The cDNA clone (GenBank accession number **HM208338**) obtained from the brain and spinal cord cDNA library and by 5′ RACE amplification is 2,073 bp long, and its longest open reading frame codes for a protein of 116 amino acid residues with a predicted molecular mass of 12.9 kDa (data not shown). There are two initiator methionine codons at the 5′ end and a stop codon at the 3′ end. In addition, the 3′ untranslated region (UTR) contains four polyadenylation tails. Therefore, the cDNA encodes a full-length sequence protein.

**Figure 1:**
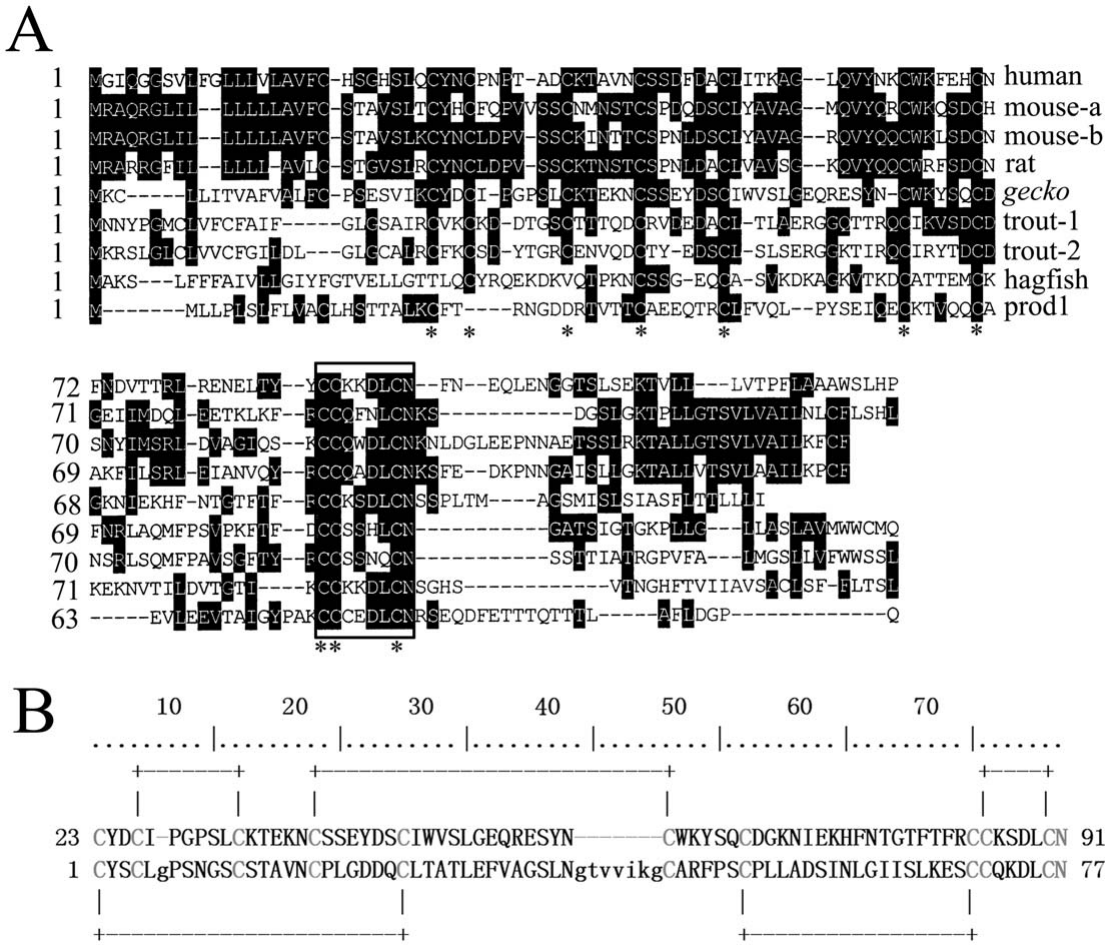
The deduced amino acid sequence of gecko *CD59* aligned with those of several mammals and newt Prod1. (A) Shaded (with solid black) residues are the amino acids that match the consensus sequence. Gaps introduced into sequences to optimize alignment are represented by dashes. The asterisk represents the ten cysteines residues that are conserved among members of the family. The residues enclosed in a box indicate the conserved CD59/Ly-6 family motif CCXXXXCN. (B) Alignments of gecko CD59 with pfam00021, the typical sequence of CD59 family. The conserved and active sites are marked by gray color and the predicted disulfide bonds are also indicated by lines. Sequences obtained from GenBank or SwissProt are gecko (HM208338), human (NP_000602.1), mouse-a (NP_031678), mouse-b (NP_862906), rat (AAH63176), trout 1 (AY593999), trout 2 (AJ880383), hagfish (AAD47892), Prod1 (ABV29331).

The PSI-BLAST searching at NCBI showed that the deduced protein shared 37% (40/108) identity to human CD59 sequence (NP_000602.1). An alignment of the amino acid sequence with CD59 proteins and pfam00021 (Figure 1A and B), the target domain from the CDD database (UPAR_LY6, u-PAR/Ly-6 domain), showed that the protein had the conserved CD59/Ly-6 family motif CCXXXXCN. Moreover, prediction by the DiANNA program demonstrated that it possessed five pairs of disulfide bonds (Figure 1B), characteristic of human CD59 protein (PDB ID: 2UX2). All the results suggested that the encoded protein was a member of CD59 family. Gecko CD59 shares 15.1%, 19.8%, 20.7%, 21.6%, 25.0%, 25.9%, 24.1% and 26.7% identity with newt Prod1, hagfish, trout, rat, mouse and human CD59, respectively.

**Figure 2:**
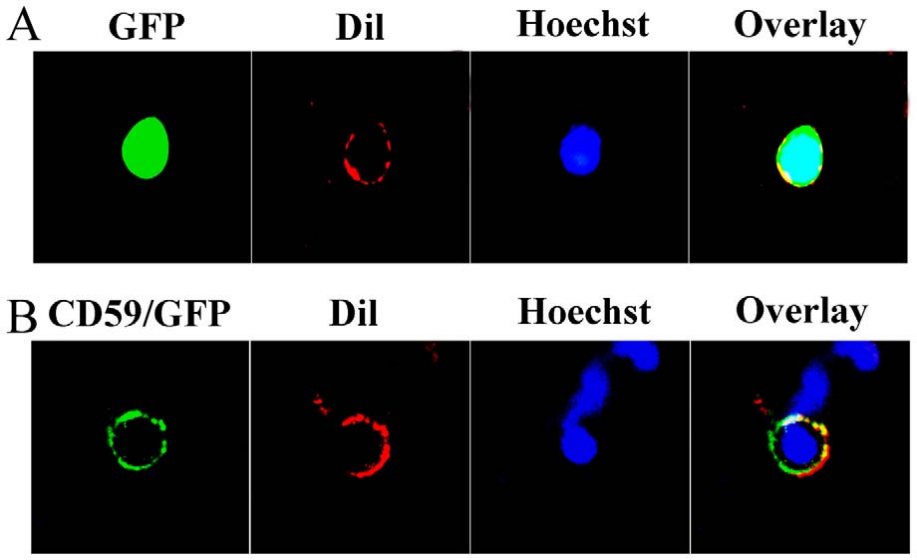
Analysis of the cellular localization of CD59 protein in transfected cells. (A) 293T cells transfected with pEGFP-N3 plasmids showing expression in cytoplasm and nucleus. (B) Colocalization of CD59 and DiI dye in the cell membrane at 7 days after transfection with pEGFP-N3-CD59 plasmids, counterstaining with the Hoechst 33342.

To present evidence of surface localization, plasmid- pEGFP-N3-CD59 and pEGFP-N3 vector were transiently transfected into 293T cells. Cells were counterstained with Hoechst 33342 (1 µg/ml), and DiI dye, which has previously been shown to embed in the lipid bilayer of cell membranes [23]. The results demonstrated that the recombinant protein colocalized with DiI, providing clear evidence for the surface expression (Figure 2). 293T cells transfected with pEGFP-N3 vector showed an expression of both cytoplasm and nucleus (Figure 2).

### Different gradient of *CD59* expression in the spinal cord and tail

**Figure 3:**
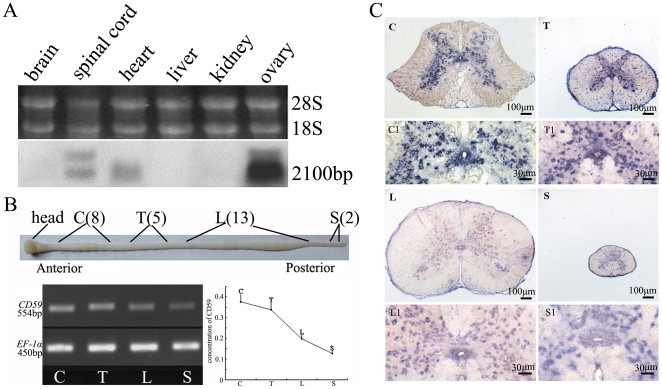
Tissue expression of gecko *CD59*. (A) Northern blotting analysis of *CD59* transcripts in different gecko tissues. The bands indicate the position of molecular size equivalent to 2,100 bp. (B) Semi-quantitative RT-PCR analysis of *CD59* transcripts in spinal cord along the anterior-posterior axis. Gecko *EF-1α* was used for normalization. C, cervical segment, from 1^st^–8^th^ cervical vertebra; T, thoracic segment, from 1^st^–5^th^ thoracic vertebra; L, lumbar segment, from 1^st^–13^th^ lumbar vertebra; and S, sacral segment, from 1^st^–2^nd^ sacral vertebra. (C) Expression of *CD59* in spinal cord was analyzed by *in situ* hybridization. C1, T1, L1 and S1 are magnifications of C, T, L and S, respectively. Scale bars: C-S, 100 µm; C1-S1, 30 µm.

**Figure 4:**
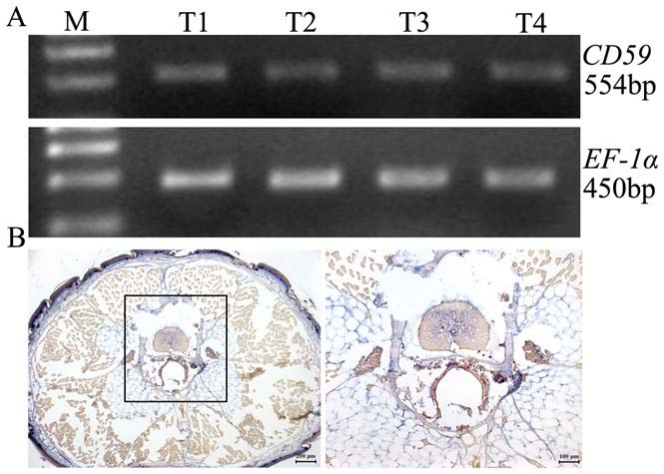
*CD59* expression in the normal tail. (A) RT-PCR amplification of *CD59* for four average segments of the tail. (B) Expression of *CD59* in the tail was analyzed by *in situ* hybridization. Positive signals in the gray matter and ependymal cells are indicated on the micrographs.

Northern blotting was conducted to assess the size of the transcript and its tissue distribution. As shown in Figure 3A, an approximately 2,100 bp band of *CD59* transcript was detected in the spinal cord, heart and ovary, although a positive signal was not seen for brain, liver and kidney. To better understand the role of gecko CD59 on tail regeneration, considerable attention was then concentrated on the *CD59* expression in the spinal cord and tail. *CD59* transcripts in an adult gecko spinal cord were detected by preparing four segments along the anterior-posterior (AP) axis (i.e. cervico, thoracic, lumbar and sacral segment, refer to Material and Methods), and were normalized to endogenous *EF-1α*. RT-PCR results revealed that gecko *CD59* displayed a graded expression in an adult gecko spinal cord, with the highest level in the anterior segment and the lowest level in the posterior (Figure 3B). Sections taken at different segments were cut and processed together on the same slides, and hybridized with DIG-labeled gecko *CD59* riboprobe. *CD59* positive signal was distributed in the gray matter with faint expression in the ependymal cells (Figure 3C). In a normal tail detached from the sixth caudal vertebra, and averagely cut into four segments, *CD59* transcript levels presented no changes between each sample. It was found that positive signals were mainly distributed in the spinal cord (Figure 4A and 4B).

### Responses of *CD59* to the tail amputation

**Figure 5:**
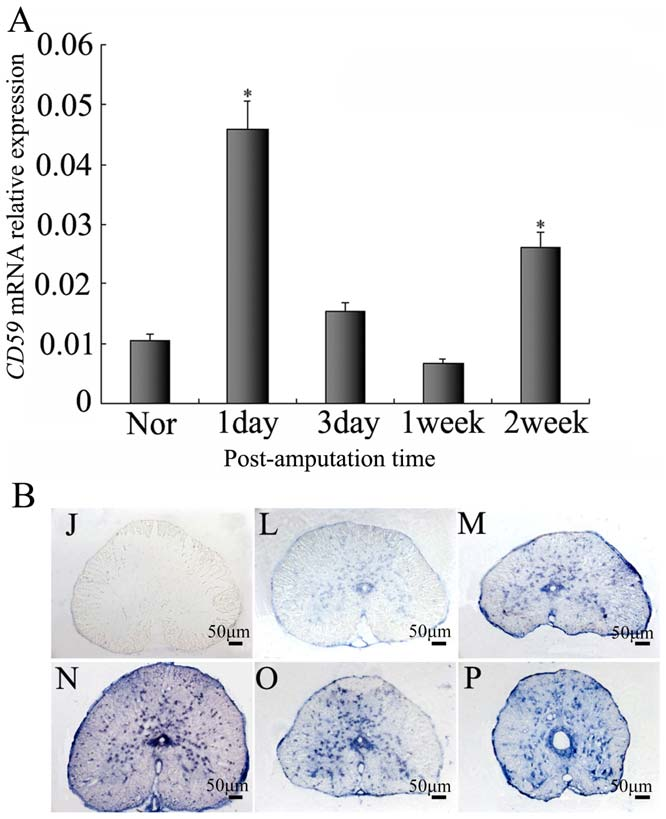
Real-time PCR analysis of *CD59* expression in the regenerating spinal cord. (A) Quantitative results for RT-PCR amplification of *CD59* for the spinal cord from L13 to the 6^th^ caudal vertebra for the controls (Nor) and following tail amputation at 1 day, 3 days, 1 week and 2 weeks. Quantities were normalized to endogenous *EF-1α* expression. Error bars represent the standard deviation (P<0.01). (B) Localization of *CD59* mRNA in the spinal cord by *in situ* hybridization using *CD59* antisense RNA probes. L, M, O and P indicate sections of spinal cord segments at 1 day, 3 days, 1 week and 2 weeks post amputation. J indicates control section with sense probe. Scale bars: J-P, 50 µm.

The total RNAs of the gecko spinal cord following amputation from L13 to the sixth caudal vertebra were used for quantitative real time PCR assay to investigate changes in the expression of the *CD59*. Real time PCR revealed that gecko *CD59* transcripts increased markedly at 1 day, but subsequently decreased at 3 days until 1 week. Interestingly, the significant increase of *CD59* mRNA was seen at 2 weeks (Figure 5A). *In situ* hybridization presented no differential *CD59* distribution in the spinal cord sections (Figure 5B). Results demonstrated that CD59 generated responses to tail amputation in the following regeneration.

### Mediation of CD59 in the engulfment of PD blastemas

**Figure 6:**
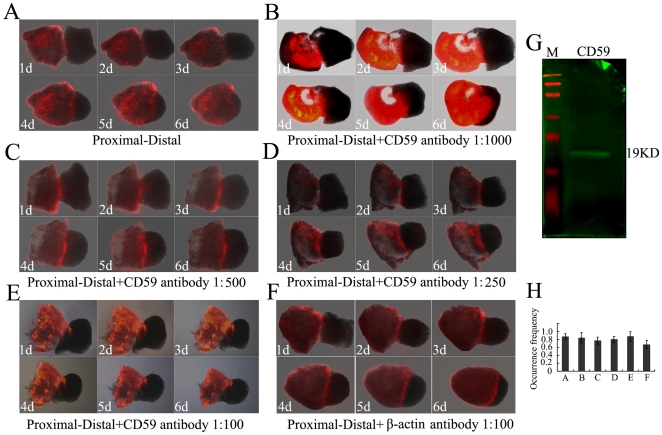
Analysis of PD blastemal confrontations in culture. (A) Engulfment behavior of blastemal confrontation cultures. The time course of engulfment is shown by sequential images at 1 day to 6 days. The proximal blastema is labeled with a tracker DiI. (B), (C) and (D) Coculture in the presence of human CD59 antibodies at dilution 1∶1000, 1∶500 and 1∶250, respectively. (E) PD engulfment was blocked co-culturing with rabbit-anti human CD59 antibody at 1∶100. (F) Coculture of a proximal with a distal blastema in the presence of rabbit-anti human β-actin antibody at 1∶100. The β-actin antibody did not generate an inhibitory effect. (G) Cross reaction of rabbit-anti human CD59 antibody with gecko blastema was analyzed by western blot. (H) Occurrence frequency of the PD engulfment in A–F.

PD confrontation assays were performed for PD identity in the tail blastemal cells, following da Silva et al. [17]. The epidermis was removed from tail regenerates at different PD levels at 20 days after amputation, and the mesenchymal tissue was juxtaposed in paired PD conjugates and cultured for 6 days. After tail amputation in the lizard, the blastema stage usually begins at the end of the fifth day with a standard cone of blastemal cell formation, and differentiation of cells in blastema sustained for at least 30–40 days [24], [25]. The proximal blastema was dyed with a tracker DiI. After 48 hours in culture, the PD pair already showed engulfment behavior with cells from the proximal partner migrating over the distal, and after 144 hours, the proximal partner engulfed the distal (Figure 6A). No migration was observed in PP or DD combinations (data not shown). To address a direct role of CD59 protein by antibody perturbation, we added affinity-purified rabbit-anti human CD59 antibody to the culture medium of PD confrontations at serial dilution. The antibody showed cross-reaction with the CD59 antigen of gecko examined by western blot (Figure 6G). When the antibody was added at 1∶100 dilution, none of the experimental pairs showed engulfment up to 144 hours in culture (Figure 6E). While the antibodies were added at dilution from 1∶250–1∶1000 (Figure 6B–6D), engulfment proceeded progressively. The occurrence frequency in each engulfment experiment was shown in Figure 6H. These findings demonstrated that rabbit-anti human CD59 antibody was active in blocking PD engulfment. The control experiments added rabbit-anti human β-actin antibody at 1∶100 dilution and did not generate inhibitory effects (Figure 6F). The data strongly suggest that CD59 is likely to be implicated in PD identity in tail blastemal cells.

### Up-regulation of *CD59* expression in tail blastema treated with RA

**Figure 7:**
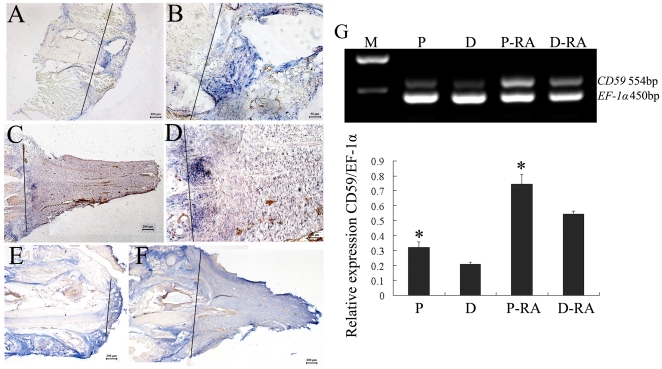
*In situ* hybridization analysis of the *CD59* expression in the blastemas. Geckos were amputated and injected with DMSO (A–D) or RA (E–F). (A) showing the expression of *CD59* in the blastemas at 1 week post amputation and (C) at 20 days post amputation; (B) and (D) indicating high power view of (A) and (C), respectively. (E) showing the expression of the *CD59* in the blastems of RA-treated animals at 1 week post amputation and (F) 20 days post amputation. (G) RT-PCR analysis of CD59 transcripts in the 20-day blastemas of DMSO-treated or RA-treated animals. Each blastema was cut averagely prior to RNA extraction. Quantities were normalized to endogenous *EF-1α* expression. Error bars represent the standard deviation (P<0.01). Line marks the plane of amputation. P, proximal segment of blastema; D, distal segment of blastema, P-RA, proximal segment of blastema treated with RA; D-RA, distal segment of blastema treated with RA. Scale bars: A, C, E, F, 200 µm; B, D, 50 µm.

In order to determine if RA regulates the expression of *CD59* during regeneration, the geckos were injected post-amputation with either RA or dimethylsulfoxide (DMSO). In DMSO-treated animals, *CD59* transcripts were distributed in the forming blastema at 1 week, with strong positive signals in proximal dedifferentiated cells and moderate staining in cells under the wound epithelium (Figure 7A and 7B). At 20 days post-amputation, *CD59* transcript was strongly detected along the boundary of amputation, and gradually decreased along the PD axis of the blastema (Figure 7C and 7D). After intraperitoneally injected with 100 RA µg/g body weight post-amputation (the dose was proved to be at the range of effective concentration by our previous experiments [9]), *CD59* transcripts in blastemas increased in comparison with the counterparts. In 1- week blastemas, positive staining presented in the center along the original medula spinalis, in the blastemal cells as well as in the muscle tissues (Figure 7E). *CD59* positive signals in 20-day blastemas were detected in all of the tail buds and muscle tissues anterior to the plane of amputation (Figure 7F). RT-PCR analysis also revealed that both 20-day blastemas treated with DMSO or RA and cut averagely, showed a gradient along the proximodistal axis (Figure 7G). It was evident that *CD59* expression was up-regulated after RA injection relative to the untreated blatemas (Figure 7G).

### Proximalizaiton of blastema cells by overexpression of *CD59*

**Figure 8:**
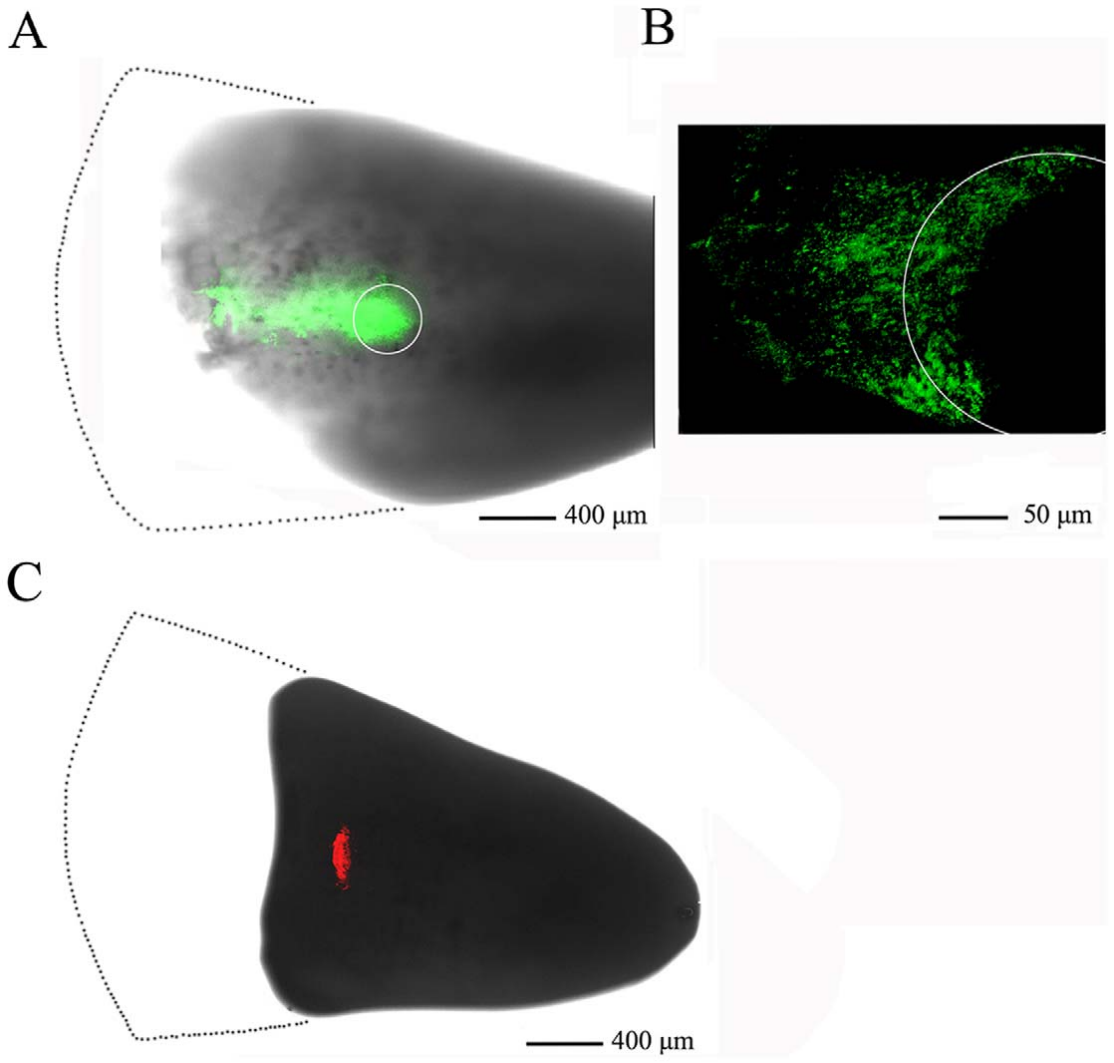
CD59 overexpression induces proximalization of blastemal cells. (A) 20-day blastema electroporated with pEGFP-N3-CD59 plasmid at 3 days post-electroporation. White circle indicates the plasmid-injected site; (B) Dorsal view of the (A); (C) Parallel experiment electroporated with pDsRed-monoter-C1 was used as a control. Dot line area indicates part of the blastema cut away for better capture of image.

To ascertain if CD59 resets the positional identity of blastemal cells *in vivo*, 20-day blastemas were chosen to electroporate with pEGFP-N3-CD59 in the middle zone, while electroporation of pDsRed-monoter-C1 was used as a control. When *CD59* was overexpressed in the blastemal cells for 3 days, the transfected cells shifted proximally (Figure 8A and 8B). In the control experiments, the marked cells remained in the region of plasmids-microinjected sites (Figure 8C). The results indicate that CD59 mediates proximodistal cell identities.

## Discussion

Several components of the immune system have been found to be involved in the regeneration process. Complement components C3 and C5, for example, are expressed in the blastemal cells of the regenerating urodele limb and lens, and implicated in the process of dedifferentiation and regeneration [26], [27]. As such, the roles of immune factors on tissue and/or organ regeneration have received much attention. We have cloned a CD59 cDNA from *Gekko japonicus* using EST analysis and RACE-PCR techniques. The full-length cDNA sequence encodes a protein of 116 amino acid residues, with a consensus sequence motif of CD59/Ly-6 family- CCXXXXCN- at C-terminal, and five pairs of disulfide bonds predicted by the DiANNA program. Gecko CD59 presents surface localization, and shares 15.1% identity with newt Prod1 in amino acid sequence, while 24.1%–26.7% identities with mammalian CD59 proteins. The expression profile showed that gecko *CD59* transcripts were detected in limited tissues, such as spinal cord, heart and ovary, compared to the broad tissue expression in vertebrates that acted to protect them from homologous complement-mediated destruction [28]–[30]. The expression variation in the gecko possibly implies different functions for the protein.

The engulfment of distal blastemas by proximal ones indicates the existence of a graded distribution of cell surface adhesive properties along the PD axis [12], [17]. The molecule involved in PD identity in the cell surface is able to sense the disparity between blastemal cells, thus mediating cellular properties such as proliferation, adhesion and migration, which may be important in tissue patterning. The engulfment assay for the gecko tail blastemas was blocked by rabbit anti-human CD59 antibody, rather than anti- gecko CD59 itself, suggesting that gecko CD59 is a significant factor in PD identity during tail regeneration. Overexpression of *CD59* in the blastemal cells re-specifies the transfected cells to a more proximal location, providing direct evidence that CD59 is implicated in PD identity *in vivo*. The data could be more conclusive if additional confrontation assays in the urodele limb were referenced. The hypothesis, however, cannot be substantiated due to the heterogeneity of transplanted tail blastema. During the tail regeneration, *CD59* transcripts were up-regulated by RA, indicating that the positional information of tail regeneration was also mediated by RA, similar to that found in limb regeneration.

There are several models to describe how blastemal cells arising on the PD axis derive their levels of expression after amputation. Kumar et al. [10] considered two possibilities. One is that the cells experience different concentrations of RA along the axis, and this sets the appropriate initial level of Prod1. The other is that Prod1 is expressed in a stable gradient along the axis in the cells of the adult limb that are precursors of blastemal cells, and that after amputation the blastemal cells inherit this level of expression. The regeneration of amphibian limbs is consistent with the latter model. Unlike urodele Prod1, gecko *CD59* transcripts kept stable along the normal tail, albeit the spinal cord from the 1^st^ cervical vertebra to the 2^nd^ sacral vertebra, showed an A>P gradient of expression. Evidently, the precursors of blastemal cells are unlikely to inherit the expression level from the tail and establish PD identity, as found in urodele limb regeneration. A gradient expression of *CD59* in the adult spinal cord and aggregation at the amputation site implied that blastemal cells derived PD identity from the spinal cord as in the first model mentioned above, but the question needs to be further addressed.

Whether regeneration of tissues or organs in extensive species is implicated in the positional identity of blastemal cells, or if they even share the common determinant of PD cell identity, remains unclear. *Gekko japonicus* has the ability to regenerate its tail from any level of amputation, and the process of regeneration is a continuous one. Following amputation, blood along with other body fluids and necrotic cellular material formed a clot, which dries into a hard wound scab by day one and two. At the margins of the broken surface underneath the scab and beyond the open end of the central canal, lymphocytes, macrophages and microglial elements of the spinal cord accumulated. By day three, melanocytes were found on the dorsal surface of the spinal cord. On the fourth day, an outgrowth of the ependyma appeared as a sac or vesicle. Wound epithelium became much thicker to form a thick cap, and a cone of blastemal cells developed on the fifth day. Two days later, within the blastema, the promuscle aggregates appeared. At 10–11 days, a wide space was still observed between the regenerating myoblasts and the original musculature, occupied by a loose mesenchyme. By days 11–14, the ependyma approached the epithelium and the regenerate began to enlarge. When the regenerate became 1 mm in length, the apical region, occupied by blastemal cells, began to form an ordered aggregate around the outgrowing ependymal tube. Over the next few days, chondroblasts formed the cartilaginous tube, and other tissues including scale began to develop [24]. Blastemal cells in the tail regeneration of *Gekko japonicus* should obtain positional instruction to achieve a correctly patterned regenerated structure. By comparison with solution structures of other three-finger protein (TFP) superfamily, Garza-Garcia et al. [19] revealed that Prod1 and its role in encoding PD identity was restricted to salamanders. The lack of comparable limb-regenerative capability in other adult vertebrates could be correlated with the absence of the Prod1 gene. The findings of CD59 and its role in PD identity during gecko tail regeneration suggested that positional information was not restricted in the urodele, but also occurred in reptiles. The Prod1 and gecko CD59 showed a functional consistency in positional identity during limb and tail regeneration, respectively. So far as we know, there were no reports on the CD59 implicated in the positional information of both vertebrates and mammals [30]–[34]. How the gecko CD59 retains this distinct function during phylogeny deserves further study.

## Materials and Methods

### Animals

Adult *Gekko japonicus* were used as described by Jiang et al [35]. Briefly, adult animals were fed freely with mealworms and housed in an air-conditioned room under controlled temperature (22–25°C) and saturated humidity. Anesthesia was induced by cooling the animals on ice prior to tail amputation. Amputation was performed at the sixth caudal vertebra, identified based on the special tissue structure present at that position [36], by placing a slipknot of nylon thread and pulling gently until the tail was detached, thus mimicking the conditions of tail loss that the animals undergo in the natural environment.

For RA treatment, all-trans RA (Sigma) was dissolved in DMSO and injected intraperitoneally (100 µg/g body weight) post-amputation according to the protocol of Mercader et al. [37]. Control experiments were performed by the injection, after amputation, of an equivalent amount of DMSO without RA.

All experiments were conducted in accordance with guidelines established by the NIH, found in *Guide for the Care and Use of Laboratory Animal* (1985), and by the Society for Neuroscience, found in *Guidelines for the Use of Animals in Neuroscience Research*. The experiments were approved according to the Animal Care and Use Committee of Nantong University and the Jiangsu Province Animal Care Ethics Committee (Approval ID: SYXK(SU)2007-0021). All geckos (n = 15) were anaesthetized on ice prior to euthanatizing, and the animal carcasses were disposed together by the animal center.

### Cloning and analysis of cDNA

A cDNA library of the brain and spinal cord from *Gekko japonicus* was constructed according to methods described previously [38]. In a large scale sequencing of the cDNA library, more than 5,000 clones were analyzed for coding probability with the DNATools program [39]. To obtain the full length of gecko *CD59*, anti-sense primer 5′-TGCAGCACCTAAACGTAAAGGTACCGGTG-3′ was designed according to the partial cDNA sequence, and 5′ RACE was performed using the BD SMART RACE cDNA Amplification Kit (Clontech, USA) according to the manufacturer's instructions. Comparison against the GenBank protein database was performed using the PSI-BLAST network server at the National Center for Biotechnology Information [40]. The protein sequence was then aligned using the BLASTP program against the CDD database to seek the possible conserved domain [41]. The disulfide bonds prediction was performed by DiANNA 1.1 software [42] using the conserved protein sequence of 23 to 91 amino acid residues of gecko CD59. Multiple protein sequences were aligned using the MegAlign program by the CLUSTAL method in the DNASTAR software package [43].

### Transfection and immunocytochemistry

293T cells were placed in 24-well plates with glass slides for transient transfection. Plasmid- pEGFP-N3-CD59 and pEGFP-N3 vector were transiently transfected into 293T cells by Lipofectamine™ 2000 (Invitrogen) following the manufacturer's instructions. At 7 days after transfection, cells were counterstained with the Hoechst 33342 (1 µg/ml) and DiI dye (Sigma, 0.05 mg/ml PBS) for 10 min at 37°C in the dark. Images were captured on a Nikon Diaphot microscope.

### RNA isolation and polymerase chain reaction (PCR)

Total RNA was prepared with Trizol (Gibco, USA) from different tissues, including the brain, spinal cord, heart, liver, kidney and ovary of adult geckos. Total RNA was also extracted from spinal cord segments: the cervico segment, included a region of spinal cord from the 1^st^–8^th^ cervical vertebra; thoracic segment, from 1^st^–5^th^ thoracic vertebra; lumbar segment, from 1^st^–13^th^ lumbar vertebra and sacral segment, from 1^st^–2^nd^ sacral vertebra. For detection of *CD59* expression along the normal tail, the tail was cut into four average segments and total RNA was isolated.

For semi-quantitative reverse transcriptase polymerase chain reaction (RT-PCR), the first-strand cDNA was synthesized using the Omniscript Reverse Transcription Kit (QIAGEN) in a 20 µL reaction system containing 2 µg total RNA, 0.2 U/µL M-MLV reverse transcriptase, 0.5 mM dNTP mix, 1 µM Oligo-dT primer. A 1 µL aliquot from the synthesized first-strand cDNA was amplified with antisense primer 5′- CAAAGATGCTATGCTGAGGG -3′ and sense primer 5′ - GCCAAGTGGAAAGCGACG -3′ designed to investigate the expression of *CD59*. The PCR amplification reaction was performed in 1.5 mM MgCl_2_, 200 µM of each dNTPs, with 20 pmol of each primer and 2 U of Taq polymerase (MBI Fermentas) in a final volume of 50 µL. After the cDNA/primer denaturation at 94°C for 3 min, the PCR amplification was carried out in 29 cycles using the following parameters: denaturation at 94°C for 30 s, annealing at 55°C for 30 s, elongation at 72°C for 30 s. The reaction was continued for a final extension at 72°C for 10 min. Normalization was carried out simultaneously by amplification of *EF-1α* using an antisense primer 5′-CTGGCTGTAAGGTGGCTCAG-3′ and a sense primer 5′-CATGTCGATTCTGGCAAGTC-3′ under the same conditions described above. A negative control without the first-strand cDNA was also performed. The expression levels were assessed by an image analysis system.

For quantitative real-time polymerase chain reaction (Q-PCR) to detect *CD59* expression changes proximal to the amputation sites, methods were used as described in Wang et al. [44]. Total RNA was prepared from the vertebra segment containing a region of spinal cord extending from L13 to the 6^th^ caudal vertebra. The first-strand cDNA was synthesized and diluted 1∶5 before use in Q-PCR assays. The primers and Taqman probe targeting conserved sequences were designed and synthesized by Invitrogen (Shanghai, China). The sequences of the primer pair and probe were: forward primer 5′-CAGTCGCAGGCTGCTCTGTATA-3′, reverse primer 5′- GACACTTCATCTTCTCGTTTGTTATGC -3′ and taqman probe 5′- CCCTCGGGCAGCGGCAGCGT -3′. Q-PCR reactions were performed in a final volume of 20 µl (1 µl cDNA template and 19 µl Q-PCR reaction buffer containing 2.5 mmol/L MgCl_2_, 0.2 mmol/L dNTPs, *CD59* anti-sense and sense primers 0.5 µmol/L, *CD59* taqman probe 0.4 µmol/L, DNA polymerase 0.2 µl and 1×DNA polymerase buffer). The Rotor-Gene 5 software (Corbett Research, Rotor-Gene, Australia) was used for real-time PCR analysis. Reactions were processed using one initial denaturation cycle at 93°C for 2 min followed by 40 cycles of 93°C for 30 s, 62°C for 30 s and 72°C for 30 s. Fluorescence was recorded during each annealing step. At the end of each PCR run, data were automatically analyzed by the system and amplification plots obtained. A *CD59* full-length plasmid was used to prepare standard curves and used as a specificity control for real-time PCR. The expression levels of the *CD59* cDNA were normalized to an endogenous *EF-1α* cDNA using forward primer 5′-CCTTCAAATATGCCTGGGT-3′, reverse primer 5′-CAGCACAGTCAGCTTGAGAG-3′ and taqman probe 5′-TTGGACAAGCTGAAGGCAGAACGTG-3′. Additionally, a negative control without the first-strand cDNA was performed. The statistical analyses were performed using STATA 7.0 software.

### Northern blotting

Northern blotting was performed as described by Wang et al. [9]. Digoxigenin (Dig)-labeled gecko *CD59* riboprobes of about 600 bp were synthesized *in vitro* from linearized plasmid following the DIG-UTP supplier's instructions (Roche). The antisense riboprobe was complementary to the 3′ end of gecko *CD59* cDNA. 4 µg of total RNA extracted from brain, spinal cord, heart, liver, kidney and ovary of adult geckos was electrophoresed and blotted onto a Nylon membrane (Osmonics Inc.). The blots were hybridized at high stringency with DIG-labeled gecko *CD59* riboprobe of about 600 bp (1 µg/ml in DIG Easy Hyb) for 15 h at 55°C, and washed twice in 2×SSC with 0.1% SDS at 25°C for 5 min each and twice in 0.1×SSC with 0.1% SDS at 65°C for 20 min each. They were then incubated in a blocking solution (pH 7.5) containing 100 mM maleic acid, 150 mM NaCl and 1% blocking reagent (Roche, Germany) followed by incubation in the blocking solution plus anti-Digoxigenin-AP (Roche, Germany) diluted 1∶10,000, for 1 and 2 h, respectively, at room temperature. After washing with 100 mM maleic acid buffer (pH 7.5) containing 150 mM NaCl and 0.3% Tween-20 and then 100 mM Tris-HCl buffer (pH 9.5) containing 100 mM NaCl, the hybridized bands were visualized by CDP-Star (Roche, Germany) and recorded by exposure to X-ray film.

### Western blot

Gecko spinal cord (n = 15) was lysed in a buffer containing 1% NP-40, 50 mmol/l Tris pH 7.5, 5 mmol/l EDTA, 1% SDS, 1% sodium deoxycholate, 1% TritonX-100, 1 mmol/l PMSF, 10 mg/ml aprotinin, and 1 mg/ml leupeptin. After centrifugation at 12,000 r/min for 20 min at 4°C, 20 µg of total protein of each sample was loaded into a 12% SDS-PAGE gel and transferred to PVDF membranes (Millipore). The membrane was then blocked with 5% non-fat dry milk in TBS containing 0.05% Tween-20 (TBS-T) for 1 h and incubated with polyclonal rabbit anti-human CD59 IgG (ProteinTech Group, Inc. Chicago, 1∶1,500). After reaction with the second antibody, donkey anti-rabbit-IRDye (1∶10,000), the membrane was scanned with an Odyssey Infrared imager (LiCor, Lincoln, NE).

### Confrontation Assays

Blastemas (approximately 20 days post amputation) were harvested, and the epidermis was dissected away with forceps. One blastema of each pair was labeled by incubation with a DiI dye (Sigma) at a concentration of 0.05 mg/ml PBS for 15 min in serum-free media. This led to effective labeling of the outer layers of cells, which migrate during engulfment. The pairs were juxtaposed at their basal surface and cultured for 6 days in DMEM supplemented with 5% FBS at 30°C, 5% CO_2_. Rabbit anti-human CD59 IgG at serial solution or β-actin IgG was added to the culture media, the concentration corresponding to a 1∶100 dilution.

### *In situ* hybridization histochemistry

Blastemas or segments of spinal cord at desired stages were harvested and fixed overnight in 4% paraformaldehyde (pH 7.0). Tissues were then dehydrated in 10–35% W/w sucrose gradient in phosphate-buffered saline pH 7.4, and 14 µm sections from the cervico-lumbar segment or lower segment of the spinal cord were cut on a cryostat and thaw mounted onto RNase-free silicane-coated slides. The sections were prehybridized for 2 h, then hybridized with 5 ng labeled probe overnight at 42°C in a humid chamber. The signals of *in situ* hybridization were detected with a coloring solution consisting of 4.5 µg/ml NBT and 3.5 µg/ml BCIP in 100 mM Tris/HCl (pH 8.0) with 100 mM NaCl and 50 mM MgCl_2_ (Roche, Germany) in dark for 3–12 h, and then observed and photographed.

### Overexpression of *CD59*

Tail blastemas were electroporated 20 days post-amputation. Animals were anesthetized on ice and immobilized. Three µl of the plasmid pEGFP-N3-CD59 or pDsRed-monoter-C1 was injected into the mid zone of the tail blastema using a microinjector. External pulses were then applied using electrotransfer (ECM 830, Neucleofecto), 200 V, and 50 ms. Three days post-electroporation, the blastemas were cut and imaged under two-photon laser scanning fluorescence microscopy (Leica sp5).
